# Urothelial bladder carcinoma in young patients is characterized by a relatively good prognosis

**DOI:** 10.3109/03009734.2011.650797

**Published:** 2012-02-15

**Authors:** Sławomir Poletajew, Maciej Walędziak, Łukasz Fus, Paweł Pomada, Joanna Ciechańska, Aleksander Wasiutyński

**Affiliations:** Department of Pathology, Medical University of Warsaw, Warsaw, Poland

**Keywords:** Age factors, age of onset, bladder cancer, histopathology, prognosis

## Abstract

**Introduction and aim.:**

Urothelial bladder carcinoma (UBC) is a very rare condition in patients aged below 50 years. The aim of the study was to answer the question whether the characteristics of cancer in this group of patients differ from general UBC features.

**Material and methods.:**

Altogether 2160 patients treated with primary transurethral resection due to a bladder tumor were included in the study. The mean age of the cohort was 69.1 years (range 11–100). Patients were divided into three subgroups depending on age: age <41 years (group 1), age 41–50 years (group 2), age >50 years (group 3). Sex ratio, tumor grade, and stage of disease were recorded.

**Results.:**

Women constituted 18.5%, 19.2%, and 25.8% of the patients in groups 1, 2, and 3, respectively (*P* < 0.05). WHO grade 3 tumors were diagnosed in 0%, 8.5%, and 17.2%, respectively (*P* < 0.05). Non-invasive papillary carcinoma was found in 100.0%, 76.7%, and 62.7%, respectively (*P* < 0.05). The incidence of muscle-invasive bladder cancer was 0%, 11.0%, and 15.6%, respectively (*P* < 0.05).

**Conclusions.:**

Pathological characteristics of UBC are dependent on the patients’ age. Being a very rare condition, UBC in young patients is characterized by a relatively good prognosis.

## Introduction

Urothelial bladder carcinoma (UBC) belongs to the most common urological malignancies. In a great majority of cases, it affects patients in their sixth or seventh decade of life. According to data from the Polish National Cancer Registry, there were 5820 newly diagnosed bladder cancers in 2008 in Poland. Among them only 48 (0.8%) were found in patients aged below 40 years and 191 (3.3%) in patients aged 40–49 years (1). On the other hand, results of UBC treatment are still not satisfactory, which has some particular importance in the context of young patients. Despite the use of advanced surgical techniques, intravesical and systemic chemotherapy, intravesical immunotherapy as well as radiotherapy, the 5-year overall survival in recent large series of patients with muscle-invasive UBC treated with radical cystectomy is reported to be as low as 47% (2).

The impact of the age at onset on tumor biology and prognosis of patients remains controversial. However, there is still an open question as to whether tumor biology can determine the age of cancer manifestation. While previous studies did not answer definitively these important questions, we investigated our database in order to determine the features of UBC in young patients and to compare them with general bladder cancer features.

## Material and methods

### Material

**Figure 1.**
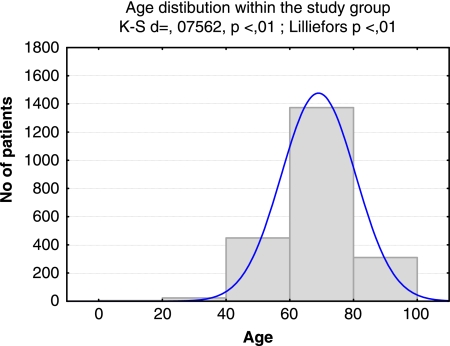
Age distribution within the study group.

Pathological records of 2160 consecutive patients with newly diagnosed UBC in the period January 2000 to June 2011 were retrospectively analyzed. The diagnosis was based on microscopic examination of the specimen obtained during primary transurethral resection of the bladder tumor. Mean age of the cohort was 69.1 years (range 11–100) (Figure 1). Women constituted 25.2% (*n* = 545) of all patients.

## Methods

Patients were divided into three subgroups depending on age: age <41 years (group 1, *n* = 27), age 41–50 years (group 2, *n* = 120), age >50 years (group 3, *n* = 2013). For all groups we assessed male-to-female ratio, tumor grade according to WHO 1973 classification, and stage of disease according to the TNM 2009 classification.

### Statistical analyses

Results are presented as numbers and percentages of patients. The differences in qualitative variables were compared with chi-square test using the Pearson formula and considered to be statistically significant when the *P* value was < 0.05. Statistical tests were performed for selected variables in the whole study population defined as three study groups and separately for comparison between each study group. When determining correlation, we used standard correlation rate formula. All calculations were performed with Statistica 9.0 software.

## Results

### Sex ratio

**Figure 2.**
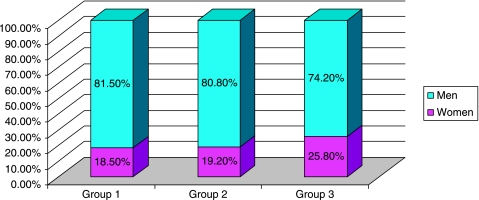
Sex distribution in the study groups.

Male-to-female ratio was calculated to be 4.4 in group 1, 4.2 in group 2, and 2.9 in group 3 (Figure 2). The differences between study groups regarding sex ratio were statistically significant (*P* = 0.046). Detailed statistical analysis showed low chi-square values and *P* values > 0.05 when comparing group 1 to groups 2 and 3, which may depend on the fact that there were just five women in group 1 (Table I).

**Table I.** Comparison of analyzed parameters between different age groups.

	Group 1 versus 2		Group 1 versus 3		Group 2 versus 3	
Parameter/Group	Chi- square	*P* value	Chi- square	*P* value	Chi-square	*P* value
Sex ratio	0.54	0.82	0.75	0.39	5.03	0.02
Grade	20.92	0.00	36.96	0.00	7.28	0.05
Stage	11.25	0.02	14.64	0.01	11.49	0.02

### Tumor grade

**Figure 3.**
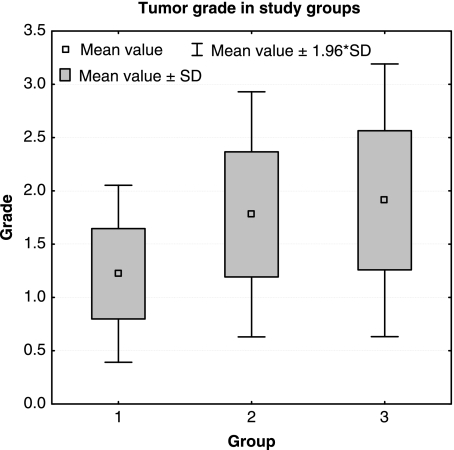
Tumor grades in the study groups.

Detailed analysis regarding the number and percentage of highly, moderately, and poorly differentiated tumors in the different study groups showed that there were substantial differences (*P* = 0.000) (Table II). We also noticed a statistically significant positive correlation between age and tumor grade (*r* = 0.126) (Figure 3).

**Table II.** Number and percentage of highly (G1), moderately (G2), and poorly (G3) differentiated tumors.

Parameter/Group		Group 1	Group 2	Group 3	*P* value
Grade (1973 WHO)	G1	21 (77.8%)	36 (30.5%)	510 (26.0%)	0.000
	G2	6 (22.2%)	72 (61.0%)	1113 (56.8%)	
	G3	0 (0%)	10 (8.5%)	336 (17.2%)	

### Stage of disease

Non-muscle-invasive UBC (Tis + Ta + T1) was found in 27 patients (100%) in group 1, in 105 patients (89.0%) in group 2, and in 1603 patients (84.4%) in group 3 (Table III). These differences between the study groups utilizing the TNM classification system were statistically significant (*P* = 0.001). This also meant that there was no muscle-invasive cancer amongst the youngest patients, whereas 11.0% (group 2) and 15.6% (group 3) of the older patients suffered from invasive (T2–T4) bladder carcinoma.

**Table III.** Stage of disease according to the TNM staging system.

Parameter/Group		Group 1	Group 2	Group 3	*P* value
Stage (2009 TNM)	Tis	0 (0%)	1 (0.8%)	46 (2.4%)	0.001
	Ta	27 (100%)	90 (76.3%)	1191 (62.7%)	
	T1	0 (0%)	14 (11.9%)	366 (19.3%)	
	≥T2	0 (0%)	13 (11.0%)	296 (15.6%)	

## Discussion

We performed a pathology analysis of UBC features in young patients and compared our results with features of UBC in a typical population, which we defined as individuals of more than 50 years of age. To the best of our knowledge, such an analysis has never been done before based on consecutive and contemporary patients, enrolling in total such significant numbers of patients younger than 50 years.

We found that the incidence of UBC in women increased with age. In patients aged <41 years the incidence was 4.5 times higher in men than in women. It was only three times higher in patients aged >50 years. In a previous investigation Shi et al. showed that there was a male-to-female ratio of 4.1 in patients aged <41 years and 2.3 in patients aged >60 years (3). Aboutaieb et al. showed that in a group of UBC patients aged <40 years there were men and women in proportions of 88.5% and 11.5%, respectively (4). However, when analyzing the paper published by Migaldi et al, the male dominance in young patients was not that clear. They noticed that women constituted 32% of patients aged <45 years and 12% amongst elderly patients (5). Some general differences between Migaldi’s and our findings may be due to the fact that they analyzed only non-muscle-invasive UBC cases, while we included all newly diagnosed UBC patients. Alanee and Shukla analyzed data from the Surveillance, Epidemiology and End Results (SEER) database maintained by the American National Cancer Institute in the context of bladder malignancies among children, showing a male-to-female ratio of 2:1. However, UBC constituted only 51% of cancers in the database. Thus, it is hard to determine the real value of this report in the context of identifying UBC features in young patients (6).

After analyzing the tumor grade in different age groups, we conclude unambiguously that age correlates with risk of presence of poorly differentiated tumors. Also the paper published by Lerena et al, which is an analysis of UBC in six children, presents a 100% incidence of low-grade carcinomas (7). Madrid Garcia et al. found that six out of eight UBC patients aged <40 years had G1 tumors (8). Fine et al. noticed the presence of low-grade tumors in 8 of 11 (73%) patients younger than 20 years (9). Low grade is one of the most important factors, underlined by Migaldi et al. and Cho et al, contributing to a better prognosis of ‘young’ patients compared to ‘elderly’ patients (5,10).

Finally, we were looking for differences in stage of the disease according to age. Our study showed a significantly higher rate of muscle-invasive UBC among patients aged >50 years compared to younger patients. After calculating the incidence of muscle-invasive UBC in patients aged <50 years, it turned out that it was almost half that of older patients. The results of a majority of previously published papers are comparable to ours (7–9,11–13). However, there are publications showing opposite results. The study by Ozbey et al. presents a 36% incidence of muscle-invasive UBC in 25 patients aged <40 years (14), while the corresponding value in the study of Aboutaieb et al. was extremely high and amounted to 58% (4). Both studies cited above were conducted in the 1990s, and the only argument for such a difference in results may be the observation of an increasing number of clinically silent and incidentally diagnosed cancers.

An additional value of our report is the statistics on the stage of UBC at the time of diagnosis in Poland. Such a report has not been published previously. While announced for the first time in Poland, its data are similar to those presented by other European investigators (15).

We used the 1973 WHO classification, which is still helpful in grading, despite the presence of more recent WHO and International Society of Urological Pathology (ISUP) classifications. This phenomenon is not unique, since many European pathologists (over 42%) still use the primary WHO classification due to its clear criteria and simplicity (16). It is also worth remembering that the clinical superiority of newer classifications compared to the 1973 WHO classification has not been confirmed (17–19).

The most important limitation of our study is the relatively small number of patients in study group 1. Even though we analyzed a 10-year-period with over 2100 cases of newly diagnosed UBC, we recruited only 27 cases of patients aged 40 or less and 147 patients aged 50 or less. Because of the changing biology of neoplasms over the past years, we believe it would not be scientifically justified to analyze longer periods of time. The solution of the problem might be a national UBC registry including pathological and clinical data, which we, however, at present lack.

Another important limitation of our study is its retrospective character. We did not re-evaluate microscopic slides, and our analysis is based exclusively on archival medical documentation. We are conscious of the fact that microscopic assessment of specimens obtained during transurethral resection of bladder tumors belongs to the most challenging in histopathology, with relatively high staging and grading errors, as well as a relatively high inter- and intra-observer variability (20–25). However, 95.2% of the slides included in our study were evaluated by the same, experienced uropathologist. A third limitation of our study might be the fact that we have not performed any clinical analysis in terms of follow-up studies. Such data would play an important role in the characterization of the biology of UBC in young patients.

Based on the results of our study, we conclude that characteristics of urothelial bladder cancer differ depending on the patients’ age. In young patients, compared to patients aged more than 50 years, cancer occurs more frequently in men, more frequently as low-grade tumors, and the disease at the time of diagnosis is more frequently at a low stage, not requiring radical cystectomy. Bladder cancer is a very rare condition in young patients; however, if it occurs, it is characterized by a relatively good prognosis.
